# Concurrent Use of Cigarettes and Smokeless Tobacco in Minnesota

**DOI:** 10.1155/2012/493109

**Published:** 2012-04-02

**Authors:** Raymond G. Boyle, Ann W. St. Claire, Ann M. Kinney, Joanne D'Silva, Charles Carusi

**Affiliations:** ^1^Research Program, ClearWay Minnesota, 8011 34th Avenue South, Suite 400, Minneapolis, MN 55425, USA; ^2^Minnesota Center for Health Statistics, Minnesota Department of Health, P.O. Box 64882, St. Paul, MN 55164-0882, USA; ^3^Behavioral Health Group, Westat, 1600 Research Boulevard, Rockville, MD 20850, USA

## Abstract

Cigarette smokers are being encouraged to use smokeless tobacco (SLT) in locations where smoking is banned. We examined state-wide data from Minnesota to measure changes over time in the use of SLT and concurrent use of cigarettes and SLT. The Minnesota Adult Tobacco Survey was conducted four times between 1999 and 2010 and has provided state-wide estimates of cigarette smoking, SLT use and concurrent use of SLT by smokers. The prevalence of SLT was essentially unchanged through 2007, then increased significantly between 2007 and 2010 (3.1% versus 4.3%, *P* < 0.05). Similarly, the prevalence of cigarette smokers who reported using SLT was stable then increased between 2007 and 2010 (4.4% versus 9.6%, *P* < 0.05). The finding of higher SLT use by smokers could indicate that smokers in Minnesota are in an experimental phase of testing alternative products as they adjust to recent public policies restricting smoking in public places. The findings are suggestive that some Minnesota smokers are switching to concurrent use of cigarettes and SLT. Future surveillance reports will be necessary to confirm the results.

## 1. Introduction

The use of snuff and chewing tobacco, commonly referred to as smokeless tobacco (SLT), has a long history of use in the United States that preceded the use of manufactured cigarettes [1]. However, use of SLT never gained the popularity of cigarettes instead it has been more common within specific subgroups such as outdoor workers and in certain geographic regions of the country. The most recent report from the Centers for Disease Control and Prevention (CDC) [2] identified key demographic characteristics associated with current use of SLT. Use was highest among males, young adults, and persons with fewer completed years of education. The CDC report provided for the first time state-specific estimates of current cigarette smokers using SLT, with the highest prevalence rates in Wyoming (13.7%) and Montana (12.1), with Minnesota also in the top tier (10.5%). The use of both cigarettes and SLT presents a unique public health challenge as concurrent users may have less desire to stop tobacco use [3] and may be less likely to quit tobacco compared to cigarette smokers [4]. This may be related to higher dependence as users of both cigarettes and SLT experience higher levels of serum cotinine [5].

In contrast to the declining sales of cigarettes [6], sales of SLT have increased in recent years [7]. However, up to this point there has been little evidence that cigarette smokers in USA switch to SLT as an alternative to cigarettes [8, 9]. We had an opportunity to examine the use of SLT among cigarette smokers as part of a tobacco surveillance system in Minnesota. The research goals for this paper are to measure changes in the prevalence rates of SLT and concurrent use of SLT by adult cigarette smokers. In addition the characteristics of smokers using SLT are described.

## 2. Methods

### 2.1. Data Source

This paper includes data from the Minnesota Adult tobacco Survey (MATS), a statewide, cross-sectional, and random digit dial (RDD) telephone survey. MATS measured tobacco use, behaviors, attitudes, and beliefs among adults aged 18 and older across 4 time points: in 1999 (*N* = 5, 968), 2003 (*n* = 8, 782), 2007 (*n* = 12, 580), and 2010 (*n* = 7, 057). After the first survey the Minnesota Department of Health Institutional Review Board reviewed and approved the 2003, 2007, and 2010 MATS questionnaire, data collection, and data security procedures.

The RDD-sampling method for all rounds of the MATS involved a two-step process; a household screening questionnaire was developed to identify households and then identify and sample people within the households. The main questionnaire contained all of the questions for the MATS adult tobacco survey interview. All rounds of the MATS used computer-assisted telephone interviewing. The survey contained the same core questions for each round and lasted between 12 and 24 minutes depending on the smoking status of the respondent.

Several communication methods were used before and during data collection for each round of the survey to improve response rates and provide information about the survey. These included letters, an informational website, and contact numbers that potential respondents could call for information. Consistent with other large-scale, telephone-based surveys, MATS telephone interviewers made a second attempt to secure cooperation by recontacting persons who initially declined to participate in the survey.

It is notable that the sample sizes for the 2003 and 2007 surveys were larger than for the other surveys. Samples of Blue Cross and Blue Shield of Minnesota members were added for these two survey years by using stratified random samples of the membership lists, then combining this sample with the RDD sample into a single file with sample weights that reflected each case's probability of selection, including the combined probability of a Blue Cross member also being sampled in the RDD sample. There was also a change in the RDD sampling frame for the 2010 survey. In order to address growing concerns about coverage in telephone surveys, the 2010 MATS used two sampling frames: (1) all possible Minnesota cellular telephone numbers, and (2) all possible Minnesota landline telephone numbers. The two samples were combined into a single file with sample weights that reflected each case's probability of selection, including the combined probability of a household with both cell and landline phones being sampled in either frame.

 The response rate for MATS 2010, calculated using the American Association for Public Opinion Research methodology, was 44.5 percent for the cell phone sample and 45.0 percent for the landline sample. These response rates are comparable to prior rounds of the MATS survey. More information on the MATS methodology can be found at http://www.mnadulttobaccosurvey.org/.

## 3. Measures

The MATS survey includes questions on demographics, tobacco use, harm perceptions, work place policies on smoking, and home policies on smoking. This paper examined variables that were asked of all users of cigarettes and SLT. Demographic questions included age, gender, marital status, and highest educational level completed. Current smokers were defined as those who reported having smoked 100 cigarettes in their lifetime and who currently smoked “every day” or “some days” at the time of the interview. Smoking intensity was categorized by the average number of cigarettes smoked per day as less than 10 cigarettes, 10 to 19 cigarettes, and a pack or more cigarettes per day.

Respondents were asked if they had used any kind of SLT, such as chewing tobacco, snuff, or snus. This categorization is consistent with the CDC's Behavioral Risk Factor Surveillance System (BRFSS). Also, respondents were asked if they thought SLT was more harmful, less harmful, or just as harmful as smoking cigarettes.

Respondents were asked in a separate question about ever use of snus, with “Camel snus or Tourney snus” as examples. This question was included because of the tobacco industry marketing that was attempting to position snus as distinct from other SLT products. For example, marketing has suggested that snus is spit-free compared to other SLTs. We defined a current snus user as using at least one day in the past 30 days.

In addition, respondents were asked how many adults who live in their household smoke. For analysis a variable was created that identified respondents (yes/no) who lived with an adult smoker. Respondents were also asked about the smoking rule in their home (not including decks, garages, or porches) “smoking is not allowed anywhere inside your home, smoking is allowed in some places or at some times, or smoking is allowed anywhere inside the home.” Those who responded that “smoking is not allowed anywhere inside” were considered to have a smoke-free home. This is comparable to how smoke-free homes are assessed in the Current Population Survey Tobacco Use Supplement (CPS-TUS).

Respondents were asked about their perception of harm from smoking an occasional cigarette (yes/no), and from breathing the smoke from other people's cigarettes. Either very harmful or somewhat harmful were combined to denote a positive response. Finally, respondents were asked if they had used any alcohol in the past 30 days (yes/no; beer, wine, wine coolers, or liquor).

## 4. Data Analysis

Statistical analyses were conducted using SPSS version 19.0 with the *Complex Samples* module. The use of this module enables the data analysis to account for the complex sample designs (e.g., multiple frames and stratification) and the sample weighting. The SPSS complex samples module uses Taylor series linearization method for estimating population characteristics [10]. The MATS surveys were weighted to represent the entire noninstitutionalized adult population in Minnesota, using raking techniques and adjustment factors to account for the dual probability of selection of cases that could have been sampled from the dual frames used in the 2003, 2007, and 2010 surveys. The resulting weights were used in the SPSS complex samples module for all analyses.

 We examined potential differences between different survey years using an analysis of nonoverlapping 95 percent confidence intervals to define significant differences. In addition statistical differences in the proportions between selected subgroups were assessed at a. 05 level based on a z-distribution. For the purpose of analysis, we created three categories of tobacco use: a current smoker (every day or some days), a current SLT user, and a concurrent user (a smoker who reported past 30 day use of SLT). For some analyses of SLT use, we also looked at former smokers who had smoked 100 cigarettes but were not currently smoking and never smokers who had not smoked 100 cigarettes.

## 5. Results

**Figure 1:**
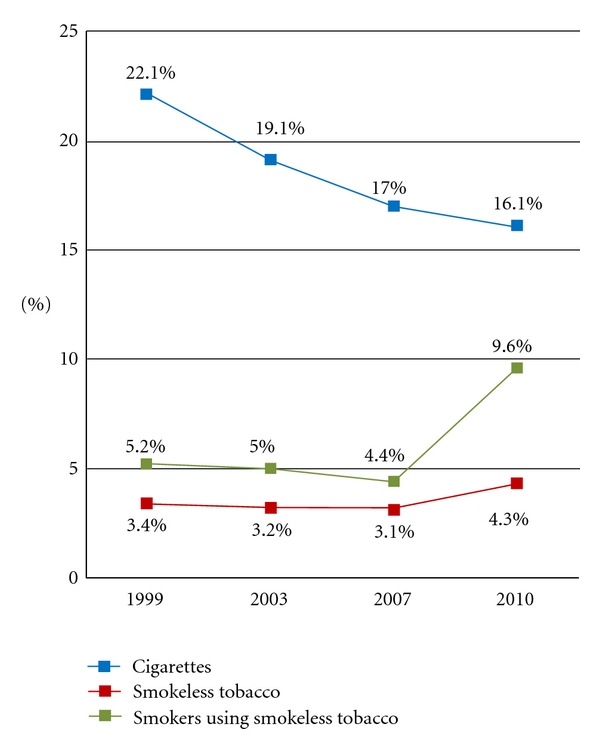
Use of cigarettes and smokeless tobacco in Minnesota, 1999–2010. The prevalence of smokeless tobacco was significantly greater (*P* < 0.05) in 2010 versus 2007. The prevalence of smokers using smokeless tobacco was significantly greater (*P* < 0.05) in 2010 versus 2007.

Figure 1 presents the prevalence of cigarette smoking, SLT use, and concurrent use of cigarettes and SLT across the four MATS surveys. Between 1999 and 2010, adult cigarette smoking prevalence in Minnesota declined from 22.1% to 16.1%, a 27.1% decrease. The rate of decrease was greatest from 1999 to 2003, and the smallest change occurred between 2007 and 2010. The state-wide prevalence of SLT was essentially unchanged through 2007, then increased significantly between 2007 and 2010 (3.1% versus 4.3%, *P* < 0.05). Similarly, the prevalence of cigarette smokers who reported using SLT was stable then increased between 2007 and 2010 (4.4% versus 9.6%, *P* < 0.05). In addition, between 2007 and 2010, there was no significant increase in the use of SLT by former smokers (3.8% versus 4.5%) or never smokers (2.4% versus 2.9%).

The use of SLT by current smokers was examined by stratifying daily and some-day smoking, and by cigarettes per day. Compared to daily smoking, some-day smokers in 2010 were significantly more likely to report use of SLT (7.3% versus 17.3%, *P* < 0.05). Light smokers (1–9 cigarettes per day) were significantly more likely to report use of SLT than smokers using half a pack or more (10-19 cigarettes) (13.7% versus 5.5%, *P* < 0.05). Smokers using a pack of cigarettes or more per day reported similar SLT use (11.1%) as light smokers.

In 2010, rates of SLT use were higher among male smokers compared to female smokers (17.8% versus 1.2%, *P* < 0.05), and among young adults ages 18 to 24 compared to adults ages 45 to 64 (24.9% versus 2.1%, *P* < 0.05). Among all Minnesota adults, the prevalence of snus use in 2010 was 1.3%; however, among smokers the rate was 3.8%. Compared to female smokers, male smokers were more likely to report use of snus (0.7% versus 6.4%, *P* < 0.05), and the highest snus use was reported by male smokers ages 18 to 24 (15.2%).

The characteristics of current smokers, SLT users, and smokers using SLT in 2010 are presented in Table 1. SLT was used almost exclusively by men (98.2%), and most concurrent users were male (93.8%). A significantly greater percentage of concurrent users (32.5%) than cigarette-only smokers (15.6%) were young adults aged 18 to 24. SLT users (91.5%) were more likely to have a home smoking ban than cigarette-only smokers (54.4%) or concurrent users (70.5%). Very few cigarette-only smokers (5%) considered SLT less harmful than smoking cigarettes; tellingly, the cigarette smokers who also used smokeless deemed smokeless as less harmful at nearly five times the rate (24.7%) of the cigarette-only group. The perception of harm from an occasional cigarette did not vary between the groups, and the perception of harm from other's cigarette smoke was high across all groups. SLT users were significantly less likely to report living with a smoker compared to smokers and concurrent users.

## 6. Discussion

This paper details recent changes in reported use of SLT products in Minnesota. A significant increase in prevalence of SLT use and smokers using SLT was observed between 2007 and 2010. Of note is the doubling in use of SLT by smokers from 2007 to 2010 whereas no similar increase was observed among former smokers or never smokers. The estimate of past 30 day use of SLT by current smokers (9.6%) is supported by current research from the CDC that found 10.5% of Minnesota smokers reported using SLT in 2009 [2].

The increase in concurrent use observed in 2010 was not predicted from earlier research. For example, Zhu and colleagues examined data from a panel of respondents in the 2002/2003 CPS-TUS and found very few men (2.2%) who smoked in 2002 but also used SLT in 2003 [8]. Others have reported low rates of concurrent use. In an analysis of earlier years of the CPS-TUS, Backinger and colleagues [11] found concurrent use of cigarettes and SLT fluctuated nationally from 3.7% in 1995 to 7.9% in 1998. Examining the period from 1992 to 2002, Mumford and colleagues reported a decline in concurrent use from the CPS-TUS [9].

There are some possible reasons that may explain the doubling of concurrent use of SLT among smokers observed in 2010. In October 2007 Minnesota implemented a comprehensive workplace indoor smoking ban that included bars and restaurants [6]. The banning of smoking indoors may have provided an opportunity for some smokers to consider smokeless alternatives to smoking.

Another reason for the increase could be industry marketing. In a recent review of SLT advertising, researchers compared advertising messages in 1998/1999 with 2005/2006. The advertising in the later period had broadened in placement and content, with more “alternative to cigarette” messages found in the later time period [12]. The increased marketing of SLT is consistent with the results from a recent review of internal tobacco industry documents that determined that cigarette manufacturers have been developing plans to provide alternatives to smokers that would offset the restrictions from smoking bans [13].

The 2009 merger of SLT and cigarette manufacturers coincided with the introduction of new SLT products in regional markets [14, 15] and nationally [16, 17]. Camel Snus, for example, was launched nationally in early 2009 and Marlboro Snus in early 2010. The introduction of these products included direct marketing to smokers. In addition, the SLT industry had previously introduced a spit-less snuff, Revel, aimed at adult smokers [18] and had been actively promoting smokeless products as alternatives, for example, promoting SLT as a substitute for cigarettes when travelling by plane (http://www.trinketsandtrash.org/).

Others have noted the advertising by the tobacco industry to encourage cigarette smokers to use SLT products as “situational substitutes” when smoking is prohibited by smoke-free air laws [14, 19]. Thus, the presence of a new indoor smoking ban, the increased advertising for SLT products, the introduction of new SLT products, and the marketing of those products to smokers may have collectively reached a receptive audience among Minnesota smokers between 2007 and 2010.

The finding of increased use of SLT products, however, represents one point in time and may not be a harbinger of future trends. Similarly, the 2010 survey was the first MATS to include a separate question about use of snus. The highest snus rate (15.2%) was among male current smokers ages 18 to 24. In another recent survey an estimated 29% of young adult male smokers reported trying snus sometime in the previous year [20]. Together these estimates suggest some receptivity by young men to the alternative product message that was promoted by the tobacco industry before and during the introduction of snus to the US market. Future surveillance will be required to determine the patterns of use and the situations in which smokers use SLT.

Our finding of higher concurrent use of SLT among younger smokers is consistent with other national surveys [3, 5]. McClave-Regan and Berkowitz [3] used a large consumer survey to examine characteristics and beliefs of adults using cigarettes, SLT, and both products. They found that a majority of concurrent users (63.6%) considered SLT as harmful as cigarettes, and far fewer (7.5%) who believed that SLT was less harmful than cigarettes. We found a similar proportion of concurrent users who considered SLT just as harmful as cigarettes (59.7%), but about 25% who considered SLT less harmful. This perception of reduced harm has received considerable debate in the scientific community [21, 22]. But our findings present one of the challenges of harm reduction, namely, that smokers may begin using SLT as a supplement to their use of cigarettes.

There are some implications that can be drawn from the information presented here. First, there is a need to consider product regulation. The tobacco industry is continuing to evolve, and this includes the introduction of new variations of current products such as snus, and the creation of novel products such as dissolvable tobacco (orbs, strips, and sticks). The introduction of new tobacco products presents an opportunity for local and state governments to consider additional regulations. The goal of reforming tobacco product regulation is to level the playing field across all tobacco products and to apply equal taxation classifications and youth access regulations to all these products. For example, in 2010 Minnesota passed the Tobacco Modernization and Compliance Act (2010 Minn. Laws ch. 305 or Senate File 3055). This law more broadly defines a tobacco product as one that can be ingested by any means. Additional information on the new law is available from the Public Health Law Center (http://www.publichealthlawcenter.org/).

 A second implication is the effect on treatment. Users of both cigarettes and SLT may have a more difficult time quitting all tobacco as their dependence/cotinine levels may be higher [5]. State tobacco control programs, help lines, and health care providers should ask about concurrent use of SLT. If a new profile of tobacco use emerges to incorporate the concept of concurrent use then cessation programs may have to adapt to this new profile by including concurrent use as a part of program content. In addition, public campaigns to promote smoke-free places should be expanded to encourage tobacco-free places.

The findings in this paper should be interpreted in light of the reliance on self-reported information, which is subject to incomplete recall and social desirability bias. In general MATS has benefited from the use of the same set of core questions, and good response rates (>40%) using RDD methods. In addition to the 2010 data representing one point in time, the observed prevalence of concurrent use does not provide sufficient detail to determine if smokers were trying SLT or had substituted SLT for some cigarettes, for example. Future surveillance will help to determine if smokers are switching to SLT as a quitting strategy or cousing in response to environmental restrictions on smoking and the direct marketing from the tobacco industry.

## 7. Conclusion

The finding of higher SLT use by smokers in 2010 compared to 2007 could indicate that smokers in Minnesota are in an experimental phase of testing alternative products as they adjust to recent public policies restricting smoking in public places. In conjunction with smoking restrictions, the tobacco industry has shifted marketing focus to SLT as an alternative to smoking. Although the findings suggest some Minnesota smokers report using SLT, future surveillance reports will be necessary to confirm these results.

## Figures and Tables

**Table 1:** Characteristics of Minnesota smokers, smokeless tobacco users, and users of both.

	Cigarette smokers (*n* = 746)		SLT users (*n* = 133)		Concurrent users (*n* = 54)	
	%	95% CI	%	95% CI	%	95% CI
Gender						
Male	46.4%	41.8–51.0	98.2%	94.1–99.5	93.8%	76.1–98.6
Female	53.6%	49.0–58.2	1.8%	0.5–5.9	6.2%	1.4–23.9
Age						
18–24	15.6%	12.3–19.6	23.8%	16.2–33.6	32.5%	20.9–46.7
25–44	41.0%	36.5–45.7	48.6%	39.1–58.1	60.5%	46.1–73.3
45–64	37.4%	33.2–41.7	23.0%	16.0–31.8	7.0%	2.8–16.3
65+	6.0%	4.8–7.6	4.6%	2.4–8.5	0%	—
Education						
Less HS	9.5%	7.0–12.8	13.9%	7.9–23.3	6.1%	1.5–21.0
HS graduate	38.3%	33.8–43.0	20.1%	13.1–29.7	52.4%	37.9–66.5
Some college	43.3%	38.9–47.9	35.9%	27.4–45.3	33.9%	22.0–48.4
College +	8.9%	7.1–11.1	30.0%	22.3–39.2	7.6%	3.3–16.4
Smoking rule at home						
Allowed	45.6%	41.0–50.2	8.5%	4.6–15.2	29.5%	17.8–44.7
Not allowed	54.4%	49.8–59.0	91.5%	84.8–95.4	70.5%	55.3–82.2
Compared to cigarettes, smokeless tobacco is…						
Less harmful	5.0%	3.4–7.4	32.2%	24.0–41.7	24.7%	13.9–40.1
More harmful	17.4%	14.1–21.2	5.1%	2.2–11.6	15.5%	6.8–31.5
Just as harmful	77.6%	73.5–81.3	62.7%	53.0–71.4	59.7%	44.2–73.5
Used alcohol past 30 days						
Yes	64.5%	59.9–68.8	76.2%	66.4–83.8	71.9%	56.3–83.6
No	35.5%	31.2–40.1	23.8%	16.2–33.6	28.1%	16.4–43.7
Harm of occasional cigarette						
Yes	54.2%	49.6–58.8	54.7%	44.7–64.3	55.2%	40.3–69.1
No	45.8%	41.2–50.4	45.3%	35.7–55.3	44.8%	30.9–59.7
Lives with a smoker						
Yes	46.0%	41.4–50.7	18.2%	12.0–26.5	48.1%	33.8–62.7
No	54.0%	49.3–58.6	81.8%	73.5–88.0	51.9%	37.3–66.2
Harm from another person's smoke						
Yes	83.4%	79.9–86.4	91.7%	83.5–96.0	84.4%	70.2–92.5
No	16.6%	13.6–20.1	8.3%	4.0–16.5	15.6%	7.5–29.8
